# Supplementary material for: Submicroscopic malaria in pregnancy and associated adverse pregnancy events: A case-cohort study of 4,352 women on the Thailand–Myanmar border
Source: PLoS Med. 2025 Mar 4;22(3):e1004529. doi: 10.1371/journal.pmed.1004529 (PMC11878921; doi:10.1371/journal.pmed.1004529)
Supplement: S1 Text — (DOCX) [file pmed.1004529.s001.docx]

**S1 Text**

**Supporting Methods**

*Data extraction and covariates*

Maternal characteristics included age, gravidity, refugee or migrant status, date of enrolment, body mass index (BMI), smoking, literacy, twin pregnancy, pre-eclampsia/eclampsia, and preterm birth history. For mMiP, the species and timing of first infection were available.

Refugee or migrant status was determined by residence: refugees (living in Mae La camp) or migrants (living in villages or migrant settlements), distinguished because of differing malaria transmission intensities.

Underweight was defined as BMI <18.5 kg/m^2^. Ideally, BMI assessment is based on the preconception BMI but as this was not available a low BMI at any stage of pregnancy was used.

Literacy, smoking and prior preterm birth were self-reported. Prior preterm birth was confirmed with medical records at the time of first ANC visit if previous delivery was at SMRU or handheld medical records from another facility were available. Gravidity was expressed differently for different outcome analyses based on physiological mechanisms of impact: for malaria and preterm birth primigravid women were compared with multigravida women [1], while those with a gravidity of 4 or more were compared with less gravid women for the analysis of anaemia [2]. Twins were diagnosed by ultrasound. Pre-eclampsia was defined as hypertension and proteinuria documented twice at least 6 hours apart after EGA of 20 weeks. Eclampsia was defined as generalized seizure in the setting of pre-eclampsia.

References:

1. Koullali B, Van Zijl MD, Kazemier BM, Oudijk MA, Mol BWJ, Pajkrt E, et al. The association between parity and spontaneous preterm birth: a population based study. BMC Pregnancy Childbirth. 2020 Dec;20(1):233.

2. Al-Farsi YM, Brooks DR, Werler MM, Cabral HJ, Al-Shafei MA, Wallenburg HC. Effect of high parity on occurrence of anemia in pregnancy: a cohort study. BMC Pregnancy Childbirth. 2011 Dec;11(1):7.
