# Supplementary material for: Submicroscopic malaria in pregnancy and associated adverse pregnancy events: A case-cohort study of 4,352 women on the Thailand–Myanmar border
Source: PLoS Med. 2025 Mar 4;22(3):e1004529. doi: 10.1371/journal.pmed.1004529 (PMC11878921; doi:10.1371/journal.pmed.1004529)
Supplement: S2 Text — (DOCX) [file pmed.1004529.s002.docx]

**S2 Text**

**Supporting Results**

In the full cohort of pregnant women receiving care during the study period 334/12,034 (2.8%) women had mMiP detected. Of these women with mMiP, 203/334 (60.8%) women had a negative blood smear for malaria at first ANC, and therefore were relevant for this analysis. Valid samples were available for uPCR for 180/203 (88.7%). Anaemia was present at some time during pregnancy in 1,544 of the 11,901 (13.0%) women without mMiP at first ANC, and 592 (38.3%) anaemic women were included in the analysis. Non-anaemic women with no mMiP accounted for 10,156/11,901 (85.2%) of women overall, and 3,580 (35.2%) were included in the analysis (**Fig 1**).

Comparison of characteristics of included and non-included women in each category confirmed similarity (**S1 Table**), with the exception of migrant or refugee status, which was further adjusted for with the strata-specific weighting.
