# Supplementary figures and images for: Submicroscopic malaria in pregnancy and associated adverse pregnancy events: A case-cohort study of 4,352 women on the Thailand–Myanmar border
Source: PLoS Med. 2025 Mar 4;22(3):e1004529. doi: 10.1371/journal.pmed.1004529 (PMC11878921; doi:10.1371/journal.pmed.1004529)

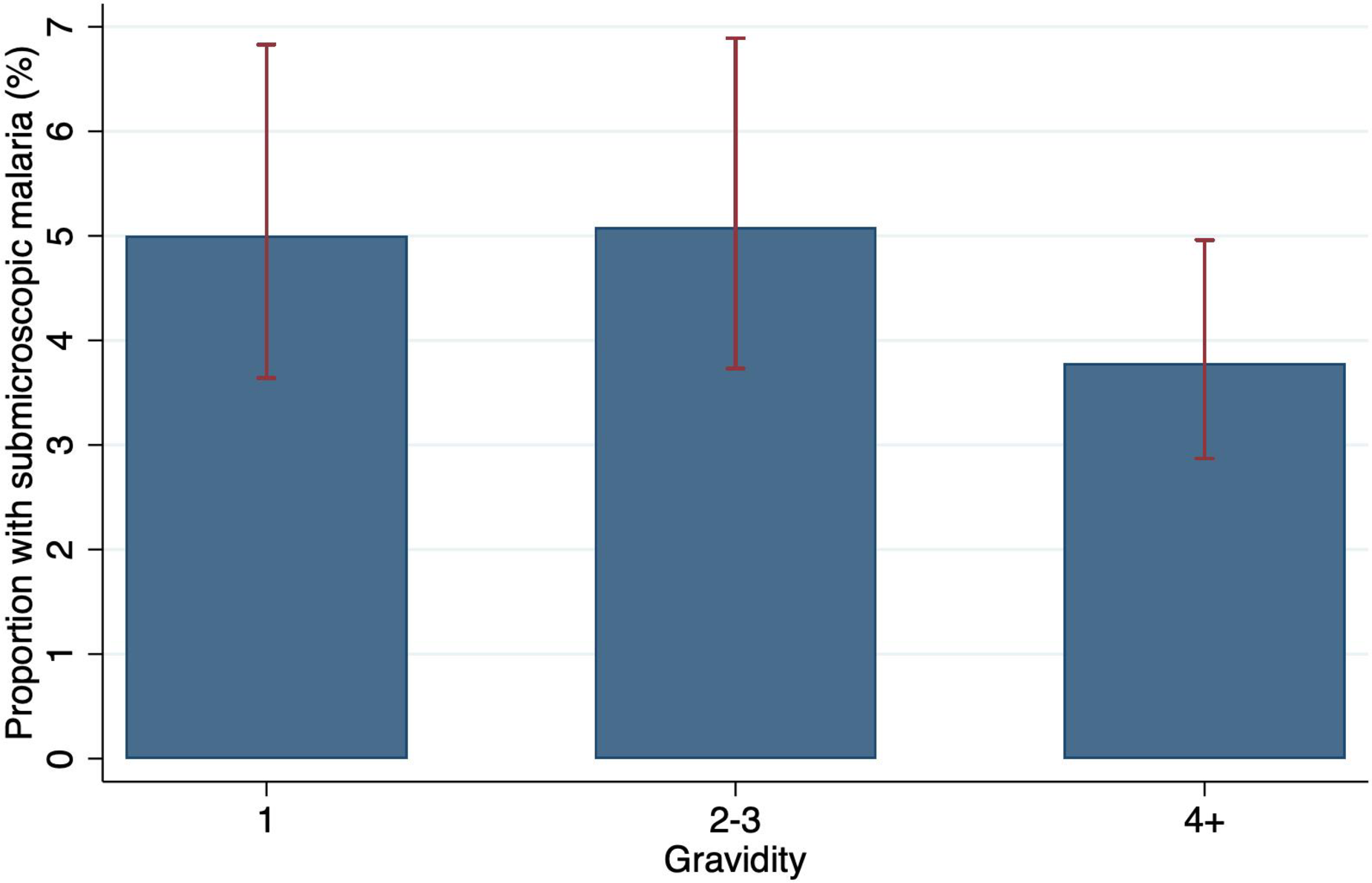

Supplement: S1 Fig — (TIF) [file pmed.1004529.s003.tif]

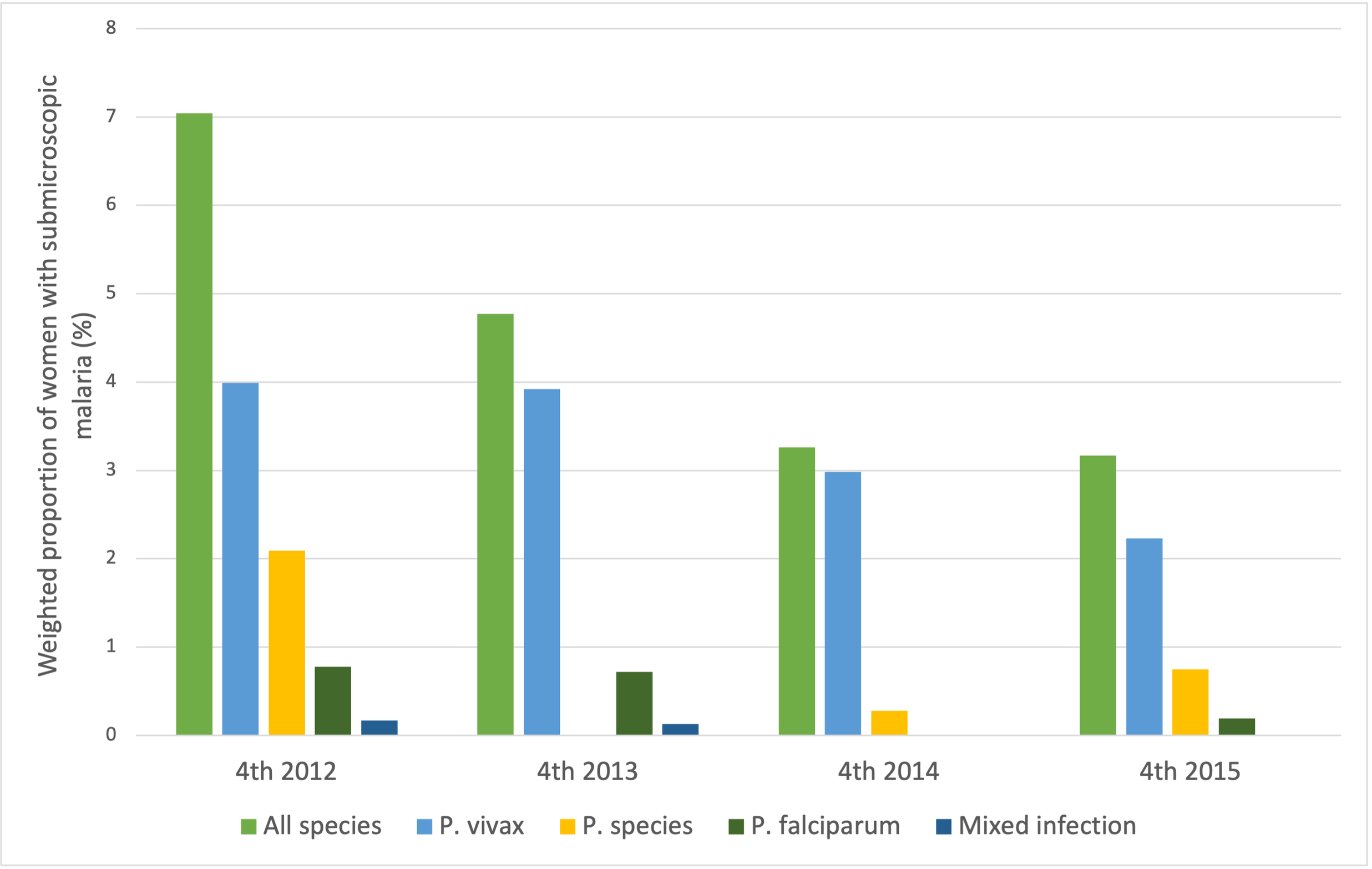

Supplement: S2 Fig — There was a substantial decrease in the weighted proportion of sMiP for all species from the 4th quarter 2012 to the 4th quarter of 2015. (Only the 4th quarter—October–December—was sampled in all 4 years.). Abbreviations: P. species, Plasmodium species (not differentiable). (TIF) [file pmed.1004529.s004.tif]

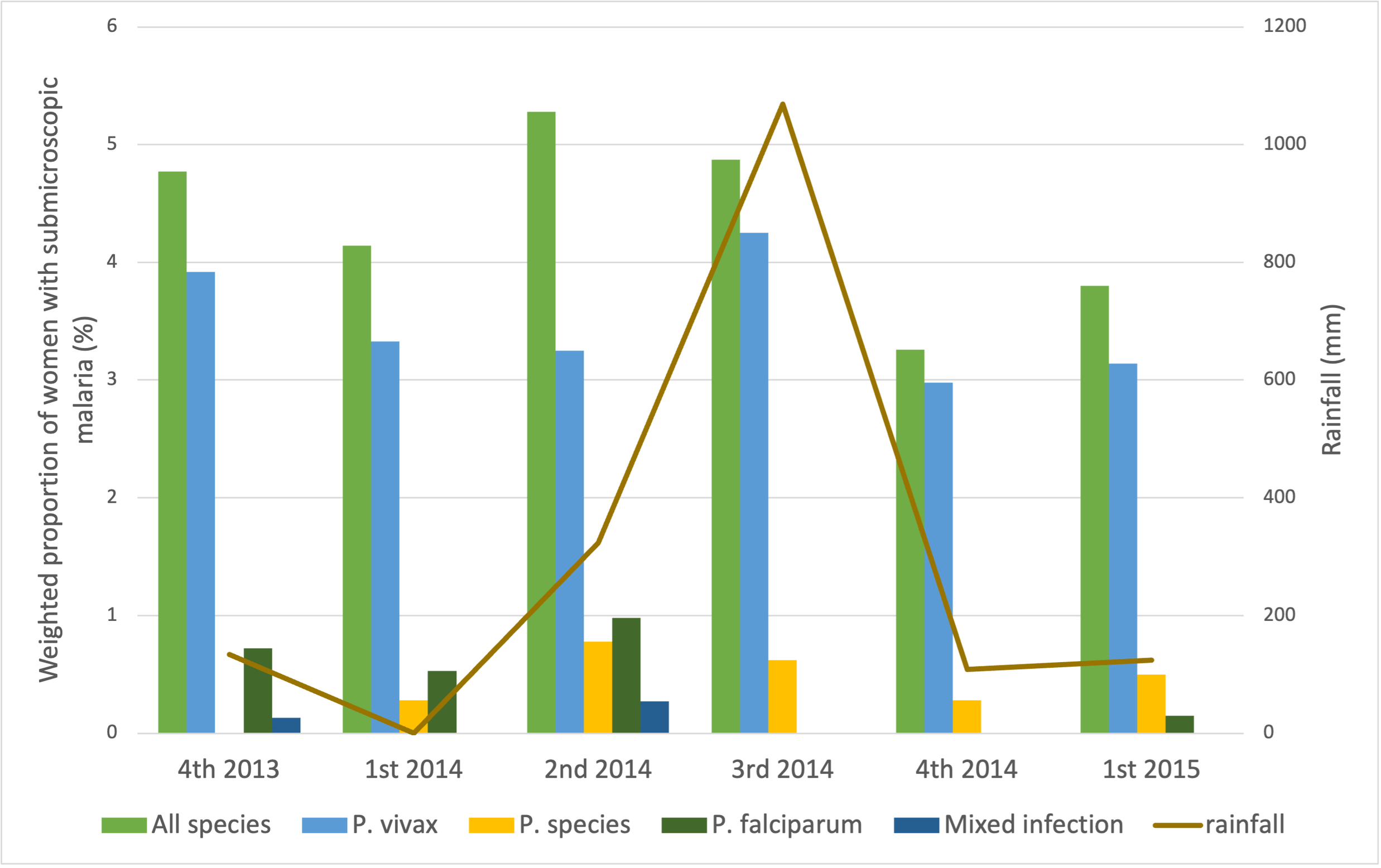

Supplement: S3 Fig — Weighted proportions of sMiP of each species were plotted against rainfall for six consecutive quarters from 4th quarter 2013–1st quarter 2015 to elucidate seasonality of sMiP. Abbreviations: P. species, Plasmodium species (not differentiable); sMiP submicroscopic malaria in pregnancy. Quarters: 1st Jan–Mar; 2nd Apr–Jun; 3rd Jul–Sep; 4th Oct–Dec. (TIF) [file pmed.1004529.s005.tif]

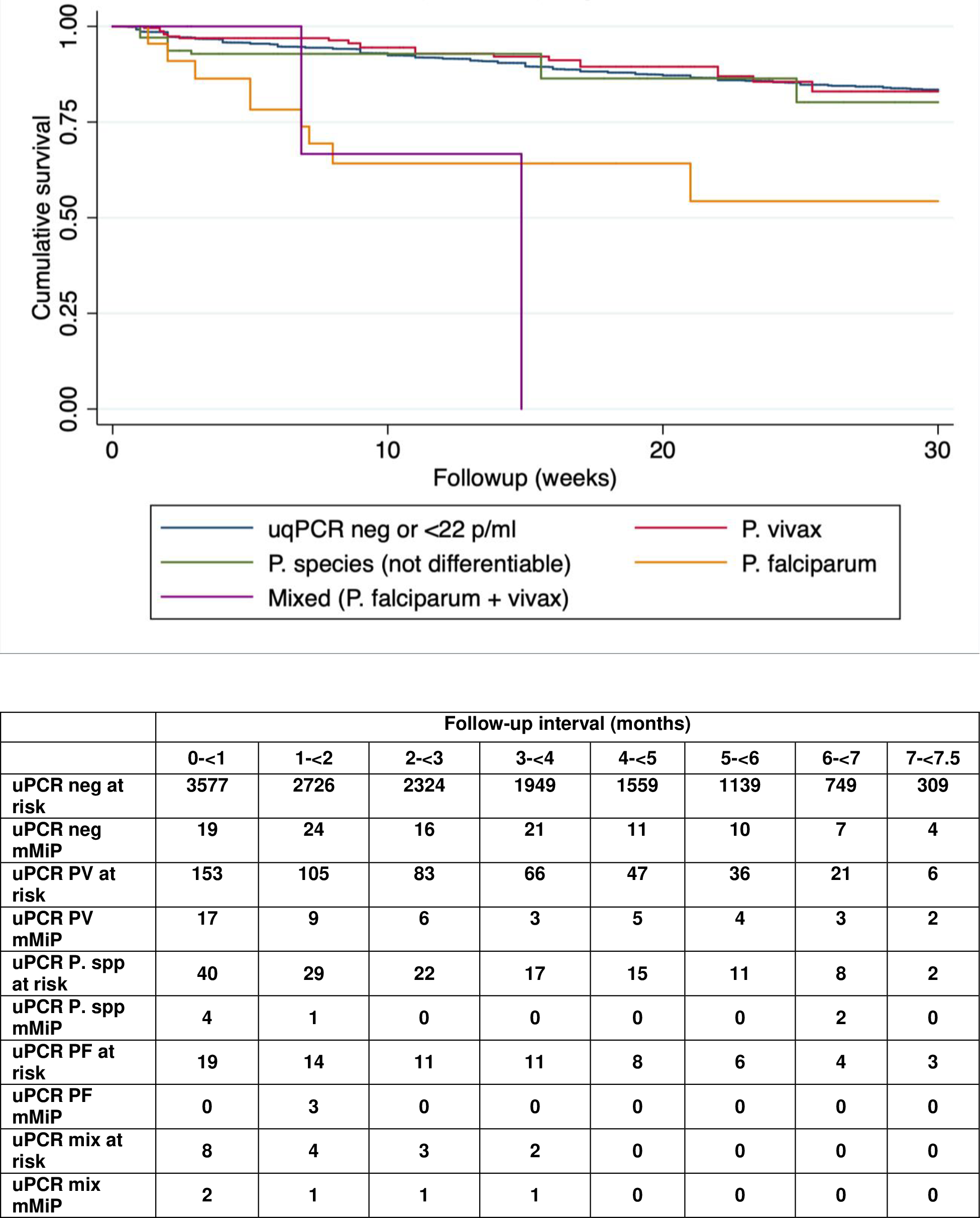

Supplement: S4 Fig — Here results are presented including mixed infection with Plasmodium falciparum and vivax. Survival terminated at 30 weeks of follow up because of sparse data beyond that time. (TIF) [file pmed.1004529.s006.tif]

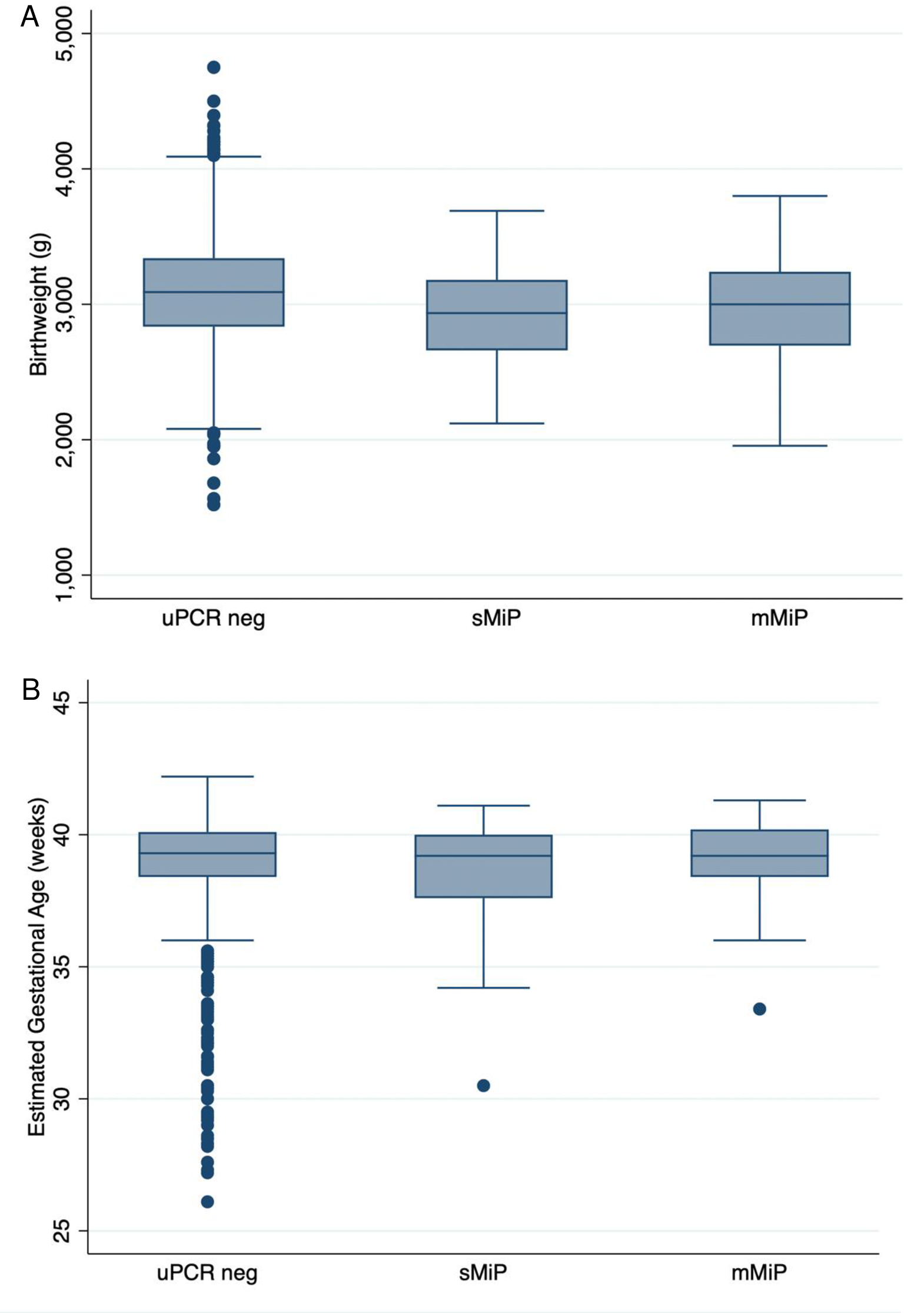

Supplement: S5 Fig — Abbreviations: uPCR, ultrasensitive quantitative polymerase chain reaction, neg, negative, sMiP, submicroscopic malaria in pregnancy, mMiP, microscopic malaria in pregnancy. Only term infants (gestational age ≥37 weeks + 0 days) were included in the birth weight figure. (TIF) [file pmed.1004529.s007.tif]
