# Supplementary material for: Submicroscopic malaria in pregnancy and associated adverse pregnancy events: A case-cohort study of 4,352 women on the Thailand–Myanmar border
Source: PLoS Med. 2025 Mar 4;22(3):e1004529. doi: 10.1371/journal.pmed.1004529 (PMC11878921; doi:10.1371/journal.pmed.1004529)
Supplement: S1 Table — (DOCX) [file pmed.1004529.s008.docx]

| **Demographics**  **(after excluding those with mMiP at first ANC)** | | Whole cohort after exclusions (n=11901) | Weighted value (n=4352) | Included Non-cases (n=3580) | Non-included Non-cases  (n=6574) | Included mMiP (n=180) | Non-included mMiP  (n=23) | Included Anaemia  (n=592) | Non-included  Anaemia  (n=952) |
| --- | --- | --- | --- | --- | --- | --- | --- | --- | --- |
| Age group | <20 | 2036 (17.1) | 16.0 | 590 (16.5) | 1139 (17.3) | 56 (31.1) | 7 (30.43) | 93 (15.7) | 151 (15.9) |
|  | 20-29 | 6033 (50.7) | 52.3 | 1857 (51.9) | 3401 (51.7) | 71 (39.4) | 12 (52.2) | 271 (45.8) | 421 (44.2) |
|  | 30+ | 3832 (32.2) | 31.7 | 1133 (31.7) | 2034 (30.9) | 53 (29.4) | 4 (17.4) | 228 (38.5) | 380 (39.9) |
| Gravidity group | 1 | 3823 (32.1) | 30.9 | 1,137 (31.8) | 2202 (33.5) | 74 (41.1) | 9 (39.1) | 155 (26.2) | 246 (25.8) |
|  | 2-3 | 4557 (38.3) | 39.4 | 1,439 (40.2) | 2546 (38.7) | 52 (28.9) | 9 (39.1) | 194 (32.8) | 317 (33.3) |
|  | >=4 | 3521 (29.6) | 29.7 | 1,004 (28.0) | 1826 (27.8) | 54 (30.0) | 5 (21.7) | 243 (41.1) | 389 (40.9) |
| Literacy | | 7191 (60.4) | 57.7 | 2,197 (61.4) | 4061 (61.8) | 91 (50.6) | 11 (47.8) | 316 (53.4) | 515 (54.1) |
| Smoking | | 1602 (13.5) | 13.3 | 469 (13.1) | 796 (12.1) | 32 (17.8) | 3 (13.0) | 103 (17.4) | 199 (20.9) |
| Migrant (not refugee) | | 7351 (61.8) | 61.8 | 1929 (53.9) | 4422 (67.2) | 150 (83.3) | 15 (65.2) | 247 (41.7) | 590 (62.0) |
| Low Body Mass Index (<18.5k/m^2^) | | 1189 (10.0) | 10.6 | 354 (9.9) | 640 (9.7) | 19 (10.6) | 6 (29.1) | 70 (11.8) | 100 (10.5) |
| First ANC in trimester 1 | | 4793 (40.3) | 41.3 | 1464 (40.9) | 2675 (40.7) | 90 (50.0) | 5 (21.7) | 253 (42.7) | 306 (32.1) |
| EGA 1^st^ ANC, weeks median [IQR] | | 17.0 [10.0-25.6] | 16.5 [9.9-25.6] | 17 [10-26] | 17 [10-25] | 13 [9-21] | 22 [17-28] | 17 [10-26] | 19 [12-27] |
| Malaria* after 1^st^ ANC | | 203 (1.7) | 1.7 | 0 (0) | 0 (0) | 180 (100) | 23 (100) | 0 (0) | 0 (0) |
| Pre-eclampsia/eclampsia | | 219 (1.8) | 1.9 | 64 (1.8) | 110 (1.7) | 4 (2.2) | 1 (4.4) | 14 (2.4) | 26 (2.7) |
| Anaemia in pregnancy | | 1,615 (13.6) | 13.5 | 0 (0) | 0 (0) | 58 (32.2) | 13 (56.5) | 292 (100) | 952 (100) |
| Haematocrit measures, median [IQR] | | 5 [2-9] | 5 [2-9] | 4 [2-7] | 5 [2-8] | 11 [7-16] | 7 [4-14] | 9 [5-13] | 8 [4-12] |
| Haemoglobin variants† | Normal or mild | 9826 (85.9) | 85.3 | 2945 (88.8) | 5517 (87.3) | 161 (91.5) | 16 (80.0) | 372 (68.4) | 680 (74.2) |
|  | Moderate | 1558 (13.6) | 14.1 | 370 (11.2) | 778 (12.3) | 15 (8.5) | 3 (15.0) | 165 (30.3) | 215 (23.5) |
|  | Severe | 59 (0.5) | 0.6 | 3 (0.1) | 22 (0.4) | 0 (0) | 1 (5) | 7 (1.3) | 22 (2.4) |
| Delivery outcome | Delivery | 8265 (69.5) | 69.0 | 2458 (68.7) | 4451 (67.7) | 151 (83.9) | 15 (65.2) | 480 (81.1) | 710 (74.6) |
|  | Twins | 97 (0.8) | 1.2 | 32 (0.9) | 42 (0.6) | 1 (0.6) | 1 (4.35) | 8 (1.4) | 13 (1.4) |
|  | Miscarriage | 879 (7.4) | 6.4 | 279 (7.8) | 502 (7.6) | 6 (3.33) | 1 (5.35) | 17 (2.9) | 74 (7.8) |
|  | Lost to follow up | 2660 (22.4) | 23.3 | 811 (22.7) | 1578 (24.0) | 22 (12.2) | 6 (26.1) | 87 (14.7) | 155 (16.3) |

**S1 Table. Characteristics of women whose samples were tested for malaria by uPCR and women who were not included, after exclusion of cases with mMiP at first ANC**

Abbreviations: ANC antenatal care, IQR interquartile range, mMiP microscopic malaria in pregnancy, Data are n (%) except for the “weighted value” column, which displays weighted percent only, unless otherwise specified
