# Supplementary material for: Submicroscopic malaria in pregnancy and associated adverse pregnancy events: A case-cohort study of 4,352 women on the Thailand–Myanmar border
Source: PLoS Med. 2025 Mar 4;22(3):e1004529. doi: 10.1371/journal.pmed.1004529 (PMC11878921; doi:10.1371/journal.pmed.1004529)
Supplement: S2 Table — (DOCX) [file pmed.1004529.s009.docx]

**S2 Table. Associations between baseline characteristics and submicroscopic malaria at first ANC**

|  | | **Weighted proportion with sMiP at 1^st^ ANC (95% CI)** | **Unadjusted Weighted OR for submicroscopic malaria (95%CI)** | **p value** | **Adjusted Weighted OR for submicroscopic malaria (95%CI)** | **p value** |
| --- | --- | --- | --- | --- | --- | --- |
| Status | Refugee | 3.0 (2.2-4.2) | Reference | - | Reference | - |
|  | Migrant | 5.7 (4.6-7.0) | 1.9 (1.3-2.9) | 0.001 | 2.0 (1.3-3.0) | 0.001 |
| Gravidity | G1 | 5.0 (3.6-6.8) | Reference | - | Reference | - |
|  | G2-3 | 5.1 (3.7-6.9) | 1.0 (0.6-1.6) | 0.942 | 1.0 (0.6-1.5) | 0.837 |
|  | G≥4 | 3.8 (2.9-5.0) | 0.7 (0.5-1.2) | 0.187 | 0.5 (0.3-0.9) | 0.012 |
| BMI  (Missing=11) | ≥18.5 | 4.8 (3.9-5.8) | Reference | *-* | *-* | *-* |
|  | <18.5 | 3.7 (2.3-5.9) | 0.8 (0.5-1.3) | 0.316 | - | *-* |
| Year of enrolment | 2012-13 | 6.0 (4.4-8.2) | Reference | - | Reference | - |
|  | 2014-15 | 3.8 (3.2-4.6) | 0.6 (0.4-0.9) | 0.015 | 0.6 (0.4-0.9) | 0.019 |
| Smoking | Non-smoker | 4.3 (3.5-5.3) | Reference | - | Reference | - |
|  | Smoker | 7.2 (5.0-10.3) | 1.7 (1.1-2.7) | 0.015 | 2.0 (1.1-3.5) | 0.017 |
| Literacy | Literate | 3.8 (2.9-4.9) | Reference | - | Reference | - |
|  | Illiterate | 5.9 (4.6-7.6) | 1.6 (1.1-2.4) | 0.015 | 1.4 (0.9-2.2) | 0.114 |
| Hb Variants  (Missing=314) | Normal/mild | 4.5 (3.6-5.5) | Reference | *-* | - | - |
|  | Moderate | 5.6 (3.0-8.1) | 1.3 (0.7-2.1) | 0.404 | - | - |
|  | Severe | 0 (0-0) | - | - | - | - |
| Fetal number | Singleton | 4.7 (3.9-5.6) | Reference | - | - | - |
|  | Twin | 3.2 (0.7-13.4) | 0.7 (0.1-3.2) | 0.619 | - | - |
