# Supplementary material for: Submicroscopic malaria in pregnancy and associated adverse pregnancy events: A case-cohort study of 4,352 women on the Thailand–Myanmar border
Source: PLoS Med. 2025 Mar 4;22(3):e1004529. doi: 10.1371/journal.pmed.1004529 (PMC11878921; doi:10.1371/journal.pmed.1004529)
Supplement: S3 Table — The microscopic species was not always the same as the antecedent uPCR malaria species at first ANC visit. Nine out of the 10 episodes of mMiP following uPCR result of P. species, were P. vivax. Median (range) time from uPCR sample to microscopically detected malaria differed by uPCR result and submicroscopic species: 77 (7–232) days for uPCR negative (n = 115), 43 (7–203) days for P. vivax (n = 49), 32 (2–223) days for P. sp. (n = 8), 49 (42–51) days for P. falciparum (n = 3), and 49 (20–105) days for mixed infections (n = 5). (DOCX) [file pmed.1004529.s010.docx]

**S3 Table. Comparison of uPCR results at first ANC among women with a subsequent positive malaria smear**

The microscopic species was not always the same as the antecedent uPCR malaria species at first ANC visit. Nine out of the ten episodes of mMiP following uPCR result of *P.* species, were *P. vivax.* Median (range) time from uPCR sample to microscopically detected malaria differed by uPCR result and submicroscopic species: 77 (7-232) days for uPCR negative (n=115), 43 (7-203) days for *P. vivax* (n=49), 32 (2-223) days for *P.* sp*.* (n=8), 49 (42-51) days for *P. falciparum* (n=3)*,* and 49 (20-105) days for mixed infections (n=5).

| **uPCR result at first ANC** | number of samples with each uPCR result | Weighted prevalence in sub-cohort,  % (95% CI) | **Microscopy diagnosed malaria species during pregnancy result** | | | | aHR† for microscopy diagnosed *P. falciparum* | aHR† for microscopy diagnosed *P. vivax* |
| --- | --- | --- | --- | --- | --- | --- | --- | --- |
|  |  |  | *P. vivax*  n (%) | *P. falciparum*  n (%) | *P. falciparum* + *P. vivax*  n (%) | Any positive*  n (%) |  |  |
| *P. vivax* | 171 | 2.8 (2.3-3.3) | 47 (30.3) | 1 (5.0) | 1 (20.0) | 49 (27.2) | 2.4 (0.2-2.5) | 17.4  (11.0-27.5) |
| *P.* species (undifferen-tiable) | 54 | 1.5 (0.9-2.4) | 7 (4.5) | 1 (5.0) | 0 (0) | 8 (4.4) | 4.6 (0.6-36.0) | 6.1 (2.5-14.7) |
| *P. falciparum* | 23 | 0.3 (0.2-0.5) | 2 (1.3) | 1 (5.0) | 0 (0) | 3 (1.7) | 33.4  (4.5-249.4) | 7.4 (1.7-32.4) |
| *P. falciparum* + *P. vivax* | 9 | 0.1 (0.0-0.2) | 1 (0.7) | 3 (15.0) | 1 (20.0) | 5 (2.8) | 278.9  (73.3-1061.0) | 16.4  (2.0-132.4) |
| Negative | 4095 | 95.3 (94.4-96.1) | 98 (63.2) | 14 (70.0) | 3 (60.0) | 115 (63.9) | reference | reference |
| Total | 4352 | 100 | 155 | 20 | 5 | 180 |  |  |

*‘Any positive’: the sum total of microscopic *P. vivax*, *P. falciparum* and Mixed species.

† adjusted for refugee/migrant status, primigravida, year of birth, smoking and literacy

Note: “*P. falciparum* + *P. vivax*” infection by uPCR indicates simultaneous coinfection (as uPCR was only done once) whereas “*P. falciparum* + *P. vivax*” by microscopy includes both simultaneous infection and/or sequential infections (e.g. *P. vivax* episode following *P. falciparum*).
