# Supplementary material for: Submicroscopic malaria in pregnancy and associated adverse pregnancy events: A case-cohort study of 4,352 women on the Thailand–Myanmar border
Source: PLoS Med. 2025 Mar 4;22(3):e1004529. doi: 10.1371/journal.pmed.1004529 (PMC11878921; doi:10.1371/journal.pmed.1004529)
Supplement: S4 Table — (DOCX) [file pmed.1004529.s011.docx]

**S4 Table. Association between submicroscopic malaria species at first antenatal care visit and birth weight z-score.**

|  | | **Weighted mean birth weight z-score (95%CI)** | **Unadjusted mean predicted difference (95% CI)** | **p value** | **Adjusted mean predicted difference (95%CI)** | **p value** |
| --- | --- | --- | --- | --- | --- | --- |
| **uqPCR category** | |  |  |  |  |  |
| Negative | | -0.44 (-0.50 to -0.38) | Reference | - | Reference | - |
| *P. vivax* | | -0.80 (-1.06 to -0.54) | *-0.36 (-0.63 to -0.10)* | *0.008* | -0.21 (-0.44 to 0.02) | 0.069 |
| *P. species (not differentiable)* | | -1.89 (-3.11 to -0.67) | *-1.45 (-2.68 to -0.23)* | *0.020* | *-1.33 (-2.43 to -0.24)* | *0.017* |
| *P. falciparum* | | -0.81 (-0.99 to -0.63) | *-0.71 (-0.56 to -0.18)* | *<0.001* | *-0.40 (-0.56 to -0.23)* | *<0.001* |
| **Status** | Refugee | -0.36 (-0.43 to -0.28) | Reference | - | Reference | - |
|  | Migrant | -0.56 (-0.65 to -0.47) | *-0.20 (-0.32 to -0.08)* | *0.001* | *-0.18 (-0.28 to -0.09)* | *<0.001* |
| **Gravidity** | Multigravida | -0.34 (-0.42 to -0.27) | Reference | - | Reference | - |
|  | Primigravida | -0.74 (-0.84 to 0.63) | *-0.39 (-0.52 to -0.27)* | *<0.001* | *-0.46 (-0.57 to -0.35)* | *<0.001* |
| **BMI** | <18.5 | -0.79 (-0.98 to -0.60) | *-0.38 (-0.58 to - 0.18)* | *<0.001* | *-0.22 (-0.35 to -0.10)* | *0.001* |
|  | ≥ 18.5 | -0.40 (-0.46 to -0.35) | Reference | *-* | Reference | *-* |
| **Year of enrolment** | 2012-13 | -0.48 (-0.61 to -0.35) | Reference | - | Reference | - |
|  | 2014-15 | -0.45 (-0.50 to -0.39) | 0.03 (-0.11 to 0.17) | 0.655 | -0.03 (-0.14 to 0.08) | 0.554 |
| **Smoking** | Nonsmoker | -0.42 (-0.48 to -0.35) | Reference | - | Reference | - |
|  | Smoker | -0.76 (-0.89 to -0.64) | *-0.34 (-0.49 to -0.20)* | *<0.001* | *-0.40 (-0.54 to -0.27)* | *<0.001* |
| **Literacy** | Literate | -0.39 (-0.46 to -0.33) | Reference | - | Reference | - |
|  | Illiterate | -0.56 (-0.66 to -0.45) | *-0.16 (-0.29 to -0.04)* | *0.011* | -0.10 (-0.21 to 0.00) | 0.059 |
| **Fetal number** | Singleton | -0.43 (-0.48 to -0.38) | Reference | - | Reference | - |
|  | Twin | -1.74 (-2.05 to -1.43) | *-1.31 (-1.62 to -0.99)* | *<0.001* | *-1.18 (-1.53 to -0.83)* | *<0.001* |
| **Pre-eclampsia or eclampsia** | Absent | -0.45 (-0.50 to -0.39) | Reference | *-* | Reference | *-* |
|  | Present | -0.84 (-1.46 to -0.23) | -0.40 (-1.01 to 0.22) | 0.204 | -0.17 (-0.55 to 0.20) | 0.364 |
| **Anaemia** | No anaemia | -0.45 (-0.51 to -0.39) | Reference | - | - | - |
|  | Anaemia | -0.50 (-0.68 to -0.31) | -0.05 (-0.24 to 0.14) | 0.609 | - | - |

N=1940. Exclusions: any woman with microscopic malaria in pregnancy, congenital abnormality, stillbirth. Abbreviations: BMI body mass index, uqPCR ultrasensitive quantitative polymerase chain reaction

* There was only one eligible neonate exposed to mixed submicroscopic infection with both *P. falciparum* and *vivax in utero*, so this neonate was excluded from the analysis*.*  (Most neonates exposed to *P. falciparum* and *vivax* submicroscopic infection at first ANC were excluded because of subsequent mMiP.)
