# Supplementary material for: Submicroscopic malaria in pregnancy and associated adverse pregnancy events: A case-cohort study of 4,352 women on the Thailand–Myanmar border
Source: PLoS Med. 2025 Mar 4;22(3):e1004529. doi: 10.1371/journal.pmed.1004529 (PMC11878921; doi:10.1371/journal.pmed.1004529)
Supplement: S5 Table — (DOCX) [file pmed.1004529.s012.docx]

**S5 Table. Association between submicroscopic malaria species and gestational age at birth (excluding women with microscopic malaria in pregnancy (mMiP))** Because of the small numbers of events in eligible records (6 preterm birth (PTB) for *P. falciparum, P.* species & mixed combined), survival analysis used submicroscopic infection with any species as the exposure.

|  | | **Weighted median EGA* (IQR)**  **(n=2291)#** | **Weighted percent with PTB (95% CI) (n=2168)†** | **Unadjusted HR of PTB (95% CI) (n=2387)^** | **p value** | **Adjusted HR**  **(95%CI)**  **(n=2387)^** | **p value** |
| --- | --- | --- | --- | --- | --- | --- | --- |
| **uPCR category** | |  |  |  |  |  |  |
| Negative | | 39.3 [38.4-40.1] | 7.2 (5.7-9.1) | Reference | - | Reference | - |
| *P. vivax* | | 39.3 [38.6-40.0] | 15.9 (4.7-42.0) | 2.3 (0.7-8.0) | 0.196 | 2.5 (0.8-7.6) | 0.095 |
| *P. species (not differentiable)* | | 38.3 [35.1-39.6] |  |  |  |  |  |
| *P. falciparum* | | 39.3 [39.0-40.0] |  |  |  |  |  |
| Mixed (*P. falciparum* & *P. vivax*) | | 37.0 [37.0-37.0] |  |  |  |  |  |
| Status | Refugee | 39.2 [38.2-40.0] | 10.6 (7.8-14.3) | Reference | - | Reference | - |
|  | Migrant | 39.3 [38.5-40.1] | 4.7 (3.4-6.3) | *0.4 (0.3-0.6)* | *<0.001* | *0.4 (0.3-0.7)* | *<0.001* |
| Gravidity | Multigravida | 39.3 [38.5-40.1] | 5.8 (4.2-7.9) | Reference | - | - | - |
|  | Primigravida | 39.1 [38.1-40.0] | 11.6 (8.2-16.0) | *2.1 (1.3-3.4)* | *0.002* | *adjusted*** | *-* |
| BMI | ≥18.5 | 39.3 [38.4-40.1] | 6.9 (5.3-8.9) | Reference | - | Reference | - |
|  | <18.5 | 39.2 [38.1-40.0] | 11.3 (6.8-18.3) | 1.7 (0.9-3.0) | 0.074 | 1.3 (0.8-2.2) | 0.347 |
| Preterm birth history | No | 39.3 [38.4-40.1] | 7.2 (5.6-9.2) | Reference | - | Reference | - |
|  | Yes | 38.5 [38.0-39.5] | 11.6 (6.5-20.1) | 1.7 (0.9-3.2) | 0.123 | 2.3 (1.1-4.8) | 0.031 |
| Year of enrolment | 2012-13 | 39.3 [38.3-40.1] | 9.2 (5.9-14.1) | Reference | - | Reference | - |
|  | 2014-15 | 39.2 [38.4-40.1] | 6.4 (5.2-8.0) | 0.7 (0.4-1.1) | 0.143 | 0.8 (0.5-1.3) | 0.360 |
| Smoking | Non-smoker | 39.3 [38.4-40.1] | 7.1 (5.5-9.1) | Reference | - | Reference | - |
|  | Smoker | 39.2 [38.2-39.6] | 10.3 (5.5-18.5) | 1.5 (0.7-2.9) | 0.281 | 1.5 (0.8-2.8) | 0.239 |
| Literacy | Literate | 39.3 [38.3-40.1] | 7.9 (5.9-10.4) | Reference | - | Reference | - |
|  | Illiterate | 39.2 [38.4-40.0] | 6.9 (4.6-10.1) | 0.9 (0.5-1.4) | 0.540 | 0.8 (0.5-1.5) | 0.566 |
| Fetal number | Singleton | 39.3 [38.4-40.1] | 6.9 (5.4-8.7) | Reference | - | Reference | - |
|  | Twin | 37.4 [36.3-38.1] | 37.0 (11.9-71.8) | 6.2 (2.1-18.2) | 0.001 | 8.9 (4.0-19.7) | <0.001 |
| Anaemia | No anaemia | 39.3 [38.3-40.1] | 7.5 (5.8-9.7) | Reference | - | - | - |
|  | Anaemia | 39.1 [38.4-39.6] | 7.2 (4.3-11.8) | 0.9 (0.5-1.7) | 0.857 | - | - |

* written as weeks.days. # included if ultrasound was done at <24 weeks, no mMiP, and they had a valid delivery date of a live, normal infant with EGA ≥24 weeks. † included if ultrasound was done at <24 weeks, no mMiP, and they had a valid delivery date of a live, normal infant with EGA >24 weeks, or were lost at ≥37 weeks EGA. ^ included if ultrasound was done at <24 weeks, no mMiP, censored when lost if lost <37 weeks EGA. Abbreviations: BMI: body mass index; EGA: estimated gestational age, HR: hazard ratio; IQR: interquartile range; P Plasmodium; PTB preterm birth; uPCR ultrasensitive quantitative polymerase chain reaction. ** Final analysis was stratified by gravidity because of violation of proportional hazards assumption for this variable. Anaemia was not adjusted for as it is on the causal pathway between sMiP and preterm birth.
