# Supplementary material for: Submicroscopic malaria in pregnancy and associated adverse pregnancy events: A case-cohort study of 4,352 women on the Thailand–Myanmar border
Source: PLoS Med. 2025 Mar 4;22(3):e1004529. doi: 10.1371/journal.pmed.1004529 (PMC11878921; doi:10.1371/journal.pmed.1004529)
Supplement: S1 Checklist — (DOCX) [file pmed.1004529.s013.docx]

STROBE Statement—checklist of items that should be included in reports of observational studies

Title: Submicroscopic malaria in pregnancy and associated adverse pregnancy events: a cohort study of 4,352 women on the Thailand-Myanmar border.

|  | Item No. | Recommendation | Section and paragraph |
| --- | --- | --- | --- |
| **Title and abstract** | 1 | (*a*) Indicate the study’s design with a commonly used term in the title or the abstract | Title |
|  |  | (*b*) Provide in the abstract an informative and balanced summary of what was done and what was found | Abstract, Author summary |
| Introduction | | | |
| Background/rationale | 2 | Explain the scientific background and rationale for the investigation being reported | Background Paragraph (P) 1-4 |
| Objectives | 3 | State specific objectives, including any prespecified hypotheses | Background P 4 |
| Methods | | | |
| Study design | 4 | Present key elements of study design early in the paper | Methods (*Study design* section) |
| Setting | 5 | Describe the setting, locations, and relevant dates, including periods of recruitment, exposure, follow-up, and data collection | Methods (*Setting, SMRU Antenatal clinic,* and *Biobanking* sections) |
| Participants | 6 | (*a*) *Cohort study*—Give the eligibility criteria, and the sources and methods of selection of participants. Describe methods of follow-up  *Case-control study*—Give the eligibility criteria, and the sources and methods of case ascertainment and control selection. Give the rationale for the choice of cases and controls  *Cross-sectional study*—Give the eligibility criteria, and the sources and methods of selection of participants | Methods (*Setting,* *SMRU Antenatal clinic, Biobanking,* and *Study design* sections) |
|  |  | (*b*) *Cohort study*—For matched studies, give matching criteria and number of exposed and unexposed  *Case-control study*—For matched studies, give matching criteria and the number of controls per case | n/a |
| Variables | 7 | Clearly define all outcomes, exposures, predictors, potential confounders, and effect modifiers. Give diagnostic criteria, if applicable | Methods (*Definition of outcomes and inclusion criteria for each outcome, Exposure,* and *Key covariate: Haemoglobin typing* sections*)* and Supplementary S1 Text |
| Data sources/ measurement | 8* | For each variable of interest, give sources of data and details of methods of assessment (measurement). Describe comparability of assessment methods if there is more than one group | Methods (Setting, *SMRU Antenatal clinic*, *Laboratory Methods, Definition of outcomes and inclusion criteria for each outcome, Exposure, Key covariate: Haemoglobin typing* sections*)* and Supplementary S1 Text |
| Bias | 9 | Describe any efforts to address potential sources of bias | Methods (*Study Design* and *Statistical methods* sections) |
| Study size | 10 | Explain how the study size was arrived at | Methods (*Study Design*) and Discussion P 5-6 |

Continued on next page

| Quantitative variables | 11 | Explain how quantitative variables were handled in the analyses. If applicable, describe which groupings were chosen and why | Methods (*Definition of outcomes and inclusion criteria for each outcome, Exposure*, *Key covariate: Haemoglobin typing* sections*)* and Supplementary S1 Text |
| --- | --- | --- | --- |
| Statistical methods | 12 | (*a*) Describe all statistical methods, including those used to control for confounding | Methods (*Statistical methods* section) |
|  |  | (*b*) Describe any methods used to examine subgroups and interactions | For subgroups, see Methods (*Statistical methods*). Interactions between confounders not tested. |
|  |  | (*c*) Explain how missing data were addressed | Methods (*Statistical methods*) P 6 |
|  |  | (*d*) *Cohort study*—If applicable, explain how loss to follow-up was addressed  *Case-control study*—If applicable, explain how matching of cases and controls was addressed  *Cross-sectional study*—If applicable, describe analytical methods taking account of sampling strategy | Methods (*Statistical methods*) P3-4 |
|  |  | (*e*) Describe any sensitivity analyses | Results (*Pregnancy outcomes: preterm birth and birth weight)* P 3 |
| Participants | 13* | (a) Report numbers of individuals at each stage of study—eg numbers potentially eligible, examined for eligibility, confirmed eligible, included in the study, completing follow-up, and analysed | Results (*Description of the cohort* section) and Figure 1 |
|  |  | (b) Give reasons for non-participation at each stage | Figure 1 |
|  |  | (c) Consider use of a flow diagram | Figure 1 |
| Descriptive data | 14* | (a) Give characteristics of study participants (eg demographic, clinical, social) and information on exposures and potential confounders | Table 1, supplementary table S1. |
|  |  | (b) Indicate number of participants with missing data for each variable of interest | Included in footnotes to tables, and Methods (*Statistical methods*) P 6 |
|  |  | (c) *Cohort study*—Summarise follow-up time (eg, average and total amount) | Methods (*Baseline characteristics at the first ANC visit in sub-cohort* section) |
| Outcome data | 15* | *Cohort study*—Report numbers of outcome events or summary measures over time | Table 1, Supplementary tables S1 and S5, Results (P1 of *Submicroscopic malaria infection at the first antenatal care visit* section, P1 of *Pregnancy outcomes: birth weight and preterm birth)* |
|  |  | *Case-control study—*Report numbers in each exposure category, or summary measures of exposure |  |
|  |  | *Cross-sectional study—*Report numbers of outcome events or summary measures |  |
| Main results | 16 | (*a*) Give unadjusted estimates and, if applicable, confounder-adjusted estimates and their precision (eg, 95% confidence interval). Make clear which confounders were adjusted for and why they were included | Results all sections, Tables 2-4, Supplementary table S5 |
|  |  | (*b*) Report category boundaries when continuous variables were categorized | Methods (*Definition of outcomes and inclusion criteria for each outcome, Exposure,* and *Key Covariate Haemoglobin Typing* sections) |
|  |  | (*c*) If relevant, consider translating estimates of relative risk into absolute risk for a meaningful time period | Hazards are presented reflecting the pregnancy period. |

Continued on next page

| Other analyses | 17 | Report other analyses done—eg analyses of subgroups and interactions, and sensitivity analyses | | Results (P2 of *Risk of subsequent microscopically detected malaria after submicroscopic infection* section, P2 of *Risk of anaemia in pregnancy with submicroscopic infection* section, and P3 of *Pregnancy outcomes: preterm birth and birth weight* section*)* |
| --- | --- | --- | --- | --- |
| Discussion | |  |  | |
| Key results | 18 | Summarise key results with reference to study objectives | | Discussion P1 |
| Limitations | 19 | Discuss limitations of the study, taking into account sources of potential bias or imprecision. Discuss both direction and magnitude of any potential bias | | Discussion P5 |
| Interpretation | 20 | Give a cautious overall interpretation of results considering objectives, limitations, multiplicity of analyses, results from similar studies, and other relevant evidence | | Discussion P6, Conclusions |
| Generalisability | 21 | Discuss the generalisability (external validity) of the study results | | Discussion P6 |
| Other information | |  |  |  |
| Funding | 22 | Give the source of funding and the role of the funders for the present study and, if applicable, for the original study on which the present article is based | | Declarations: funding |

*Give information separately for cases and controls in case-control studies and, if applicable, for exposed and unexposed groups in cohort and cross-sectional studies.

**Note:** An Explanation and Elaboration article discusses each checklist item and gives methodological background and published examples of transparent reporting. The STROBE checklist is best used in conjunction with this article (freely available on the Web sites of PLoS Medicine at http://www.plosmedicine.org/, Annals of Internal Medicine at http://www.annals.org/, and Epidemiology at http://www.epidem.com/). Information on the STROBE Initiative is available at www.strobe-statement.org.
