# Supplementary material for: Submicroscopic malaria in pregnancy and associated adverse pregnancy events: A case-cohort study of 4,352 women on the Thailand–Myanmar border
Source: PLoS Med. 2025 Mar 4;22(3):e1004529. doi: 10.1371/journal.pmed.1004529 (PMC11878921; doi:10.1371/journal.pmed.1004529)
Supplement: Attachment — Submitted filename: PlosMedResponse20241227.docx [file pmed.1004529.s018.docx]

We are very grateful for the ongoing commitment of the reviewer and editor to improve and finalize this manuscript. Please find out point by point responses below.

Editors comments:

1) Please confirm that your title complies with to PLOS Medicine's style. Your title must be nondeclarative and not a question. It should begin with main concept if possible. "Effect of" should be used only if causality can be inferred, i.e., for an RCT. Please place the study design ("A randomized controlled trial," "A retrospective study," "A modelling study," etc.) in the subtitle (ie, after a colon).

We have edited our title to more accurately reflect the type of study done: **Submicroscopic malaria in pregnancy and associated adverse pregnancy events: a case-cohort study of 4,352 women on the Thailand-Myanmar border.**

2) Please ensure that the Introduction ends with a clear description of the study question or hypothesis.

We have added the following sentence in the final paragraph of the introduction: “The objective of this study was to test if these adverse pregnancy outcomes that are associated with microscopically detected malaria infections are also associated with submicroscopic malaria detected at the first antenatal care (ANC) visit.”

3) Please ensure that all abbreviations are defined at first use throughout the text.

We reviewed the manuscript and defined additional abbreviations such as “mg” and “ml” that were not clearly defined (highlighted in the tracked changes version of the manuscript).

4) Please review your text for claims of novelty or primacy and remove this language. In addition, please check that any use of statistical terms (such as trend or significant) are supported by the data, and if not please remove them.

We have reviewed the manuscript and removed such wording, where present (e.g. the final paragraph of the introduction).

5) Please ensure that the study is reported according to the STROBE guideline, and include the completed STROBE checklist as Supporting Information. Please add the following statement, or similar, to the Methods: ""This study is reported as per the Strengthening the Reporting of Observational Studies in Epidemiology (STROBE) guideline (S1 Checklist)."

The STROBE checklist and the recommended statement have been added.

6) Did your study have a prospective protocol or analysis plan? Please state this (either way) early in the Methods section.

a) If a prospective analysis plan (from your funding proposal, IRB or other ethics committee submission, study protocol, or other planning document written before analyzing the data) was used in designing the study, please include the relevant prospectively written document with your revised manuscript as a Supporting Information file to be published alongside your study, and cite it in the Methods section. A legend for this file should be included at the end of your manuscript.

b) If no such document exists, please make sure that the Methods section transparently describes when analyses were planned, and when/why any data-driven changes to analyses took place.

c) In either case, changes in the analysis-- including those made in response to peer review comments-- should be identified as such in the Methods section of the paper, with rationale.

We did not have a specific protocol covering this analysis as the ethical approval covering this study covered an array of screening tests done at first ANC to further our understanding of anaemia in this population. We have added this information to the methods under *Study design* in the second paragraph:

“This study did not have a formal analysis plan. The preliminary analysis was planned as a cohort study. However, considering that uPCR was conducted only in a subset of the whole cohort using stratified random sampling due to cost constraints, the data was analysed as a case-cohort study using inverse sampling-probability weighting as described below based on early input from co-authors. All exposures and outcomes were pre-defined prior to analysis, while specific cut-offs for analysis were defined by the data where appropriate (e.g. parasitaemia quartiles).”

7) Your study is observational and therefore causality cannot be inferred. Please remove language that implies causality and refer to associations instead.

We have reviewed the manuscript and amended the wording to avoid statements of causality.

8) Reviewer #2: I thank the authors for their reply and updated manuscript/regression following further thought given to potential confounders. Although I believe the adjustment is sufficient the DAGs have not been constructed correctly, with multiple variables included as one, variables adjusted for but not in DAG (such as sex), adjusted variables shown rather than the total model and interactions between variables not entirely considered. However, these updates are unlikely to change the adjusted models and I therefore suggest the DAGs are removed from the figures.

We appreciate the feedback have removed the DAGs from the figures.
