# Supplementary material for: Submicroscopic malaria in pregnancy and associated adverse pregnancy events: A case-cohort study of 4,352 women on the Thailand–Myanmar border
Source: PLoS Med. 2025 Mar 4;22(3):e1004529. doi: 10.1371/journal.pmed.1004529 (PMC11878921; doi:10.1371/journal.pmed.1004529)
Supplement: Attachment — Submitted filename: 20240912uPCRreviewercomments_clean.docx [file pmed.1004529.s016.docx]

We appreciate the time and effort put into this manuscript by the editors and reviewers. Please find below our point-by-point response to the helpful comments.

Reviewer #1: This is a well written manuscript, with minimal grammatic/typo errors. In a case-cohort study, the report elegantly addresses the question if submicroscopic malaria affects inter-generational health outcomes during pregnancy.
Some points to clarify:

1) There are three groups derived from the case-cohort study, the last group regarded as "Control Group". Since the groups are derived based on outcomes, is it appropriate to designate the last group as "Control Group"? Why not just refer to it as "Group 3"?

**Response:** We have amended the manuscript to refer to this group as the “non-case” group in response to this comment.

2) For adjusting temporal changes in disease/infection prevalence, 8-time blocks were identified (lines 168-171). But the time blocks have not been characterized to show temporal trends, e.g., temporal trends of prevalence or parasitaemia. In lines 336-37, there is mention of primigravid women enrolled during early years of the case-cohort being at higher risk of getting a submicroscopic infection at their first ANC visit. This observation was not discussed considering the temporal variations.

**Response:** It is true, the analysis of temporal trends was not at the forefront of the presentation of our results. However, they are included in the supplementary figs 2&3, which show an overall decline in submicroscopic malaria over the study period. This was accounted for in the analysis by controlling for year and assigning quarter-specific weights to each result. The mention of the primigravid women was as a separate (expected) risk factor for submicroscopic malaria, alongside year of enrollment, and smoking. Given the large number of findings presented here, we were not able to go into these associations in detail. They are mentioned largely to confirm that this database showed associations between submicroscopic malaria infection and characteristics known to be associated with patent malaria.

3) Given that the uPCR has yielded parasitaemia ranges from 22 to 186,048p/ml, is there a high parasitaemic threshold above which parasitaemia is no more sMiP? In other words, is there uPCR threshold that precludes sMiP? In relation to this, can risk of developing mMiP be differentiated by parasitaemic groups: Q1, Q2, Q3, Q4? In line 250, the risk of developing mMiP, anemia and pre-term birth is given, but qualitatively based on presence or absence of sMiP.

**Response:** The reviewer highlights an important point about submicroscopic malaria – namely that it is dependent on the skill of the microscopist or the sensitivity of the RDT used to detect clinical malaria in that setting. Most manuscripts presenting data on submicroscopic malaria do not include data on the actual parasitemias observed by PCR, and many do not even clearly state the sensitivity of the assay used. Here we have attempted to be more transparent about the estimated burden of malaria indicated by a positive uPCR result, and clarify at what threshold (if any) do these infections become insignificant. This type of analysis was planned from the beginning of the research, as evidenced by the labor-intensive process of running internal standards on each uPCR run to allow estimation of parasitemia, and described in the methods, lines 231-233. Though there are some infections (as demonstrated by figure 2) that fall within or above the standard limits of detection for microscopy, these are very view and fall within expected human error rates. We did not exclude these from analysis as submicroscopic for two reasons: 1) estimation of parasite burden by qPCR also has a margin of error and 2) such infections would be included as submicroscopic infections in other reports.

4) In line 277: the given date and numbers that follow can confuse the reader. Re-writing using a combination of numbers and letters/words can help.

**Response:** Thank you for pointing this out. The line has been rephrased to enhance readability: “The SMRU ANC clinics registered and screened 12,034 women from October 1, 2012 to December 31, 2015.”

5) In lines 298-305, prevalence is given for the 3 groups: 36.1%, 5.1% and 3.8%. What was the mean parasitaemia for the 3 groups?

**Response:** This data is included in Table 1, after the breakdown by species.

6) Given that the prevalence of mixed Pf/Pv malaria infection was only 1.8%, the analysis of data on risks (e.g., risk of anemia, lines 122/123) seems overly over-interpreted. This also seen in the tables showing HR and p-values.

**Response:** We agree that it is hard to draw conclusions about the mixed infection group, given the small numbers. However, these mixed infections are encountered in clinical practice. The frequent exclusion of mixed infections from studies due to low numbers has caused a data gap on these infections, so we wanted to present what data we did have to fill this gap. We have removed some of the details from the discussion.

7) In lines 132-135 (page 31), the fact that women were closely followed, screened for malaria and treated is discussed as a limitation of the study. It seems it should rather be a strength. Perhaps changing the context will help.

**Response:** Thank you for this comment. We have expanded a little more on how this is both a strength and a weakness: “The setting of this study is both a strength and a weakness: the close follow up, repeated screening, and early treatment of microscopically detected malaria episodes may lead to an underestimation of the impact of sMiP at first ANC visit on pregnancy outcomes. This level of intense follow up is unusual in malaria endemic areas, where low-density infections may go untreated for longer periods of time. On the other hand, this intensive screening allowed this analysis to hone in on the specific impact of sMiP.”

8) In table 3, Hb variants are classified as: Normal/mild, Moderate and Severe. This seems confusing as often Hb variants are mentioned in relation to abnormal structure of globulins and heme complex (Hg S, C, D, E). Maybe it is means to describe Hgb levels?

**Response:** The information about these Hb variant categories was moved to the supplementary files. We have now brought it back to the main methods section and clarified that the categorization was based on the impact of these abnormal globulins on anaemia: “Data from Hb typing was categorized to create an ordinal variable as follows based on the impact on anaemia: normal or mild (normal or alpha thalassemia trait), moderate (Beta thalassemia trait and Hb E trait), and severe (Hb EE homozygotes, HbH, Beta thalassemia with HbE disease). Moderate and severe categories were combined when data were sparse.”


Reviewer #2: The authors present a large study examining submicroscopic malaria in pregnancy and the risk of microscopic infection, birthweight and preterm birth. They have used a pragmatic approach of a case-cohort design given the associated cost of PCR for all samples and utilised an appropriate Cox regression.


Minor comments:
♣ Postmenstrual age - should be estimated gestational age.

**Response:** amended as requested.

♣ Tables 1 is difficult to follow. Why are exclusions included in the table? This would be better suited to a flow diagram

**Response:** These lines were included to highlight the fact that a high proportion of microscopically detected malaria in pregnancy in this cohort was detected at first consultation. However, as this information is available in figure one, we agree to delete it from the table to enhance simplicity and readability.

♣ Table 1: Most data has been presented categorically including maternal age and BMI. Including a measure of central tendency (mean/median dependent on distribution of data) and spread for continuous data will provide the reader with a better understanding of the population.

**Response:** We have added BMI as a continuous variable to the table. Median and IQR for age and EGA are included in the text.

♣ Table 1: Were all data complete? If not, include number of missing for each variable

**Response:** Thank you for this suggestion. We have added this data to the footnote to Table 1.

♣ Table 1: Given the case-cohort is the population and not whole cohort, I suggest moving the column of the whole cohort (11,901) to the supplemental, this will improve the readability of the table. I understand it was included to show random sampling was effective but can be included as a supplement.

**Response:** This information has been moved to the supplement as suggested.

♣ Table 1: It is unclear why the authors have presented weighted values for maternal demographics when they have also presented adjusted analyses to account for the differences. Additionally, weighting does not appear to change proportions substantially.

**Response:** The weighted values for the demographics were included as reassurance to the reader that the weighting resulted in a good approximation of the characteristics of the full cohort. This has been moved to the supplement along with the full cohort data.

♣ Birthweight; the authors state in the methods intergrowth and z-scores were used but this does not appear to be so in the results. Adjusted analyses should consider including a measure of SES. The 95% CI for birthweight at term is very narrow, is this correct? Would expect a larger spread.

**Response:** We agree that socio-economic status is potentially an important factor. However, it has been challenging to estimate in this marginalized population, and a robust measure of SES was not established for this group. Literacy, which was included in our analyses, can be one surrogate variable reflecting SES. Z-scores were finally only included in the supplementary files as the consensus among authors was that birth weight was more interpretable. We have reviewed the analysis and the 95% CI for the mean birth weight at term was indeed, narrow, as were the CI for most groups with ~1,000 babies included. 95% CI were naturally larger for the sMiP groups which included fewer babies.

♣ Was haematocrit nadir normally distributed?

**Response:** When we applied normality tests, it was not normally distributed. We have changed the presentation (table 3 and the preceding text) to medians and IQR in response to this comment.

Major comments
♣ A significant limitation of the study is the age of the cohort, with samples collected 2012 - 2015 (ie up to 12 years old). Given the change in the population since this time, the relevance of the findings to current practice and population needs to be clarified.

**Response:** We agree that this is a limitation. However, given the continuous dynamic trends of malaria incidence in many locations, we feel the results remain relevant. We have expanded on this in the limitations: “The age of the cohort could be perceived to limit its relevance. However, the incidence of malaria in pregnancy at SMRU clinics is now more similar to 2012-2015 than it was in the intervening years, due to the increases in malaria transmission since the coup d’etat in Myanmar in 2021. This resurgence brings both microscopic and submicroscopic infections in pregnancy back to top of the maternal and child health agenda.”

In addition it adds to data for submicroscopic infections in Asia which are limited and remains relevant for other low transmission settings.

♣ Throughout the authors have used IPW models but have not provided any details of how the models were constructed. How was balance assessed and what difference was considered acceptable?

**Response:** We have added the following details about how the model was constructed and balance assessment. After IPW, all sub cohort variables differed from the full cohort by less than 3%.

" Time-fixed inverse probability weighting was used to account for differential stratified sampling probability for each group – microscopically diagnosed malaria, anaemia, and non-case [28]. Weights equal to the reciprocal of the sampling fraction for each group were calculated separately for each three to six month block, and for migrants and refugees to account for differing malaria transmission intensities over time and by location. Maternal characteristics of the sub-cohort and the prevalence of sMiP at the first ANC over time were described by weighted frequencies (proportion with 95% confidence interval) or weighted median with inter-quartile range [IQR]. Weighted characteristics of the sampled women were compared with characteristics of the cohort as a whole to assess balance. Balance was additionally checked by comparing characteristics of sampled vs unsampled participants."

♣ The authors have included selected confounders based on a p-value of <0.2. Why was this value selected? More importantly, including confounders purely based on p-values is not recommended, this can introduce bias. The use of an IPW model should consider what variables would predict being in the exposure group as well as consider confounders - not based on p-values but the use of subject knowledge and ideally, direct acyclic graphs. Were new models constructed for each maternal baseline characteristic? It is unclear which variables are included for each outcome.

**Response**: All variables included in the univariable analysis were variables that were considered linked to the outcome a priori based on previous literature and from clinical judgement, which is the reason why different models include different variables in the univariable model. For parsimony, covariates that were not associated with the outcome were removed from the final model (for example BMI for patent malaria). We can confirm that the coefficients for the main exposure was similar when we include all the potential confounders. We have expanded the methods section briefly to reflect this process better: “Confounders were conceptualized with causal models, and these potential covariates were excluded from the final multivariable models when they were not associated with the outcome (with a p value of 0.2 or higher) for parsimony. Gravidity for all outcomes, and smoking for preterm birth, were included *a priori*.”

Reviewer #3: Overall this is a very interesting publication.

minor comments- throughout the paper, all instances of microscopy detected malaria should be replaced by microscopically detected.

**Response:** Amended as requested.

Page 38, "From 2012 to 2015, approximately 1 in 22 women attending their first antenatal visit on the Thailand-Myanmar border had asymptomatic sMiP at their first antenatal care visits." - delete one of the "first antenatal care visit"

**Response:** Thank you! Amended to: “From 2012 to 2015, approximately 1 in 22 women on the Thailand-Myanmar border had asymptomatic sMiP at their first antenatal visit.”

Did you assess preterm delivery as a potential cause of some LBW?

**Response:** Yes, preterm delivery (and sex) was assumed to affect birth weight and all analysis of birth weight was therefore adjusted for gestational age and sex. As a supplementary analysis, z-score adjusted for sex and gestational age was analysed and presented as Supplementary Table 4.

I'm not entirely clear why you excluded women negative at ANC1? this could be better explained in the methods.

**Response:** Women who had a *positive* malaria smear by microscopy at first ANC were excluded in order to allow us to single out the effects of submicroscopic infection. Women negative for malaria by microscopy (or PCR) were not excluded.

Page 41- this needs a ref: "In areas of higher transmission, a higher proportion of infections are detectable by RDT or microscopy"

**Response:** Thank you for pointing this out. A citation has been added.
