# Supplementary material for: Submicroscopic malaria in pregnancy and associated adverse pregnancy events: A case-cohort study of 4,352 women on the Thailand–Myanmar border
Source: PLoS Med. 2025 Mar 4;22(3):e1004529. doi: 10.1371/journal.pmed.1004529 (PMC11878921; doi:10.1371/journal.pmed.1004529)
Supplement: Attachment — Submitted filename: Reply to statistical reviewer R2 .docx [file pmed.1004529.s017.docx]

Thank you for your valuable feedback. We have carefully considered your comments and made the following updates to our manuscript:

1. **Multivariable model construction**:

For the variables included in our final multivariable models, we agreed with the reviewer and constructed the final model based on DAGs, setting the primary exposure and the outcome. The model assessing the risk factors of submicroscopic parasitaemia at the first ANC visit was not updated as this exploratory model did not assess the causal association and there was no specific primary exposure in this model. We were interested in which factors were associated with the presence of sub-microscopic parasitaemia at the first ANC. The rest of the models were all updated.

2. **Clarification on IPW methods**:

The inverse probability weights used with the Cox regression models were not derived from a model using covariates as propensity scores, but were actually simply the inverse of the sampling fraction, by group, estimated for each three to six month block, for migrants and refugees. This approach typically does not require “covariate balance checking” beyond reporting the weighted values and providing a comparison with the whole cohort [1,2]. We have edited the methods section to clarify.

1.	Valliant R, Dever JA, Kreuter F. Practical Tools for Designing and Weighting Survey Samples. Cham: Springer International Publishing; 2018. (Statistics for Social and Behavioral Sciences).

2.	O’Brien KM, Lawrence KG, Keil AP. The Case for Case–Cohort: An Applied Epidemiologist’s Guide to Reframing Case–Cohort Studies to Improve Usability and Flexibility. Epidemiology. 2022 May;33(3):354–61.
